# Wind intensity affects fine root morphological traits with consequences for plant-soil feedback effects

**DOI:** 10.1093/aobpla/plaa050

**Published:** 2020-09-14

**Authors:** Luise Werger, Joana Bergmann, Ewald Weber, Johannes Heinze

**Affiliations:** 1 Institute of Biochemistry and Biology, University of Potsdam, Potsdam, Germany; 2 Institute of Biology, Dahlem Center of Plant Science (DCPS), Freie Universität Berlin, Berlin, Germany; 3 Berlin-Brandenburg Institute of Advanced Biodiversity Research (BBIB), Berlin, Germany; 4 Leibniz Centre for Agricultural Landscape Research (ZALF), Müncheberg, Germany

**Keywords:** Wind, root traits, root morphology, specific root length, plant–soil feedback

## Abstract

Wind influences the development, architecture and morphology of plant roots and may modify subsequent interactions between plants and soil (plant–soil feedbacks—PSFs). However, information on wind effects on fine root morphology is scarce and the extent to which wind changes plant–soil interactions remains unclear. Therefore, we investigated the effects of two wind intensity levels by manipulating surrounding vegetation height in a grassland PSF field experiment. We grew four common plant species (two grasses and two non-leguminous forbs) with soil biota either previously conditioned by these or other species and tested the effect of wind on root:shoot ratio, fine root morphological traits as well as the outcome for PSFs. Wind intensity did not affect biomass allocation (i.e. root:shoot ratio) in any species. However, fine-root morphology of all species changed under high wind intensity. High wind intensity increased specific root length and surface area and decreased root tissue density, especially in the two grasses. Similarly, the direction of PSFs changed under high wind intensity in all four species, but differences in biomass production on the different soils between high and low wind intensity were marginal and most pronounced when comparing grasses with forbs. Because soils did not differ in plant-available nor total nutrient content, the results suggest that wind-induced changes in root morphology have the potential to influence plant–soil interactions. Linking wind-induced changes in fine-root morphology to effects on PSF improves our understanding of plant–soil interactions under changing environmental conditions.

## Teaser

In a field experiment, wind intensity affected the fine-root morphology and the outcome of plant–soil interactions of grassland plants. Increasing wind intensity resulted, for instance, in increased specific root length and surface area. Similarly, growth of the grassland species on soils that either contained their own soil biota or soil biota of other species differed between wind intensities. Results suggest that aboveground environmental factors like wind induce changes in root morphology that impact plant–soil interactions. Linking wind-induced changes in root morphology to plant–soil feedback effects represents one step towards a closer understanding of plant–soil interactions under changing environmental conditions.

## Introduction

Wind is a ubiquiteous but rather neglected environmental factor that has various effects on plants (Humphries and Roberts 1965; de Langre 2008; Onoda and Anten 2011). For instance, wind affects seed dispersal (Kuparinen 2006; Pazos *et al.* 2013), photosynthesis (Ennos 1997; Clark *et al.* 2000; Burgess *et al.* 2016), leaf traits (Anten *et al.* 2010) as well as the mechanical stability of plants (Stokes *et al.* 1995; Onoda and Anten 2011; Burgess *et al.* 2016; Gardiner *et al.* 2016). Beside these aboveground effects, wind also affects biomass allocation (i.e. root:shoot ratio) and root growth (Nicoll and Ray 1996; Cleugh *et al.* 1998; Poorter *et al.* 2012; Feng *et al.* 2019). The finding that wind as a mechanical stimulus induces changes in biomass allocation is, however, mostly based on studies with woody species (e.g. Gardiner *et al.* 2016). Therefore, tests on herbaceous species are needed to obtain a more comprehensive understanding of these effects.

Wind is also known to improve the anchorage of plants by strengthening the development of roots (Danjon *et al.* 2005; Tamasi *et al.* 2005; Stofko and Kodrik 2008). Plant anchorage in soil generally depends on root system architecture (i.e. spatial configuration of the whole root system) and root morphology (i.e. characteristics of individual roots) (Ennos and Fitter 1992; Goodman *et al.* 2001; Dupuy *et al.* 2005). Although many studies investigated the effects of wind on root architecture in the context of anchorage of woody species (e.g. Stokes *et al.* 1995; Tamasi *et al.* 2005; Stofko and Kodrik 2008), studies have rarely considered root morphology (Goodman and Ennos 1997; Burylo *et al.* 2009), especially in herbaceous plants. Hence, studies that link effects of wind on root morphology in grasslands are needed.

In addition to effects on anchorage, root morphology is important for a range of interactions between plants and their abiotic and biotic soil environment (Bardgett *et al.* 2014). For example, root morphological traits have functional consequences for soil water and nutrient uptake, organic matter decomposition as well as interactions with soil biota (Mommer and Weemstra 2012; Bardgett *et al.* 2014; Smith *et al.* 2014; Minerovic *et al.* 2018; Bergmann *et al.* 2020). These processes change soil properties that in turn affect plant growth and thus plant–soil feedbacks (PSFs; see Bever 1994). Because root morphological traits are important for many soil processes they have recently gained attention in the context of plant–soil interactions and PSFs (see e.g. Bergmann *et al.* 2016; Cortois *et al.* 2016; Rutten and Gòmez-Aparicio 2018; Wilschut *et al.* 2019). These plant–soil interactions are widely influenced by many abiotic and biotic environmental drivers both directly and indirectly (Smith-Ramesh and Reynolds 2017; Bennett and Klironomos 2019). Hence, linking these drivers to plant–soil interactions could further strengthen our understanding of their impact on plant growth in changing environments (van der Putten *et al.* 2016; DeLong *et al.* 2019). Recent research suggests that aboveground biotic drivers (e.g. insect herbivory) have the potential to influence plant–soil interactions and PSFs by affecting root morphology (Heinze 2020). However, no study has directly linked wind effects on root morphology to plant–soil interactions until now. As wind was found to induce changes in root morphology in trees (Tamasi *et al.* 2005) it is likely that wind-induced changes in root morphology will influence interactions between plants and their surrounding soils as well.

The overarching goal of this research was to test the effect of wind on biomass allocation and root morphology of herbaceous plants as well as to link potential wind-induced changes in root morphology to plant–soil interactions (i.e. the outcome of PSFs). Furthermore, we aimed to test these effects under realistic wind conditions and not with electric fans in a greenhouse where natural wind is mostly excluded (Heinze *et al.* 2016; Forero *et al.* 2019). Therefore, we performed a field experiment with four grassland plant species and investigated above- and belowground biomass, root morphological traits of fine roots and PSFs at two different wind levels to answer the following questions:

1. Does wind intensity affect biomass allocation (i.e. root:shoot ratio) of grassland plants?
2. Are morphological traits of grassland plant roots altered by wind intensity?
3. Do potential wind-induced changes in root morphology influence the outcome of PSFs?

## Methods

### Study site and species

The effect of wind intensity on plant fine root morphology and plant–soil interactions was tested under field conditions in a meadow at a field site of the University of Potsdam (N52°24′29.76ʺ, E13°1′13.74ʺ, Brandenburg, Germany). The vegetation structure and abiotic and biotic conditions of this meadow have been described elsewhere (Heinze *et al.* 2016; Heinze and Joshi 2018). Briefly, average annual precipitation (550 mm) and temperature (11.5 °C) at this site varies from highest mean values in July (79 mm; 18.4 °C) to lowest mean values in January/February (35 mm; −1.2 °C). The meadow is located on nutrient poor slightly sandy loam and was low-intensity managed for the last 20 years with no fertilization. The vegetation in this meadow comprises a high plant species diversity with a mean species richness of 16 species per m^2^ (Heinze and Joshi 2018).

To test whether wind intensity affects root morphological traits of grassland plants we selected four grassland species belonging to two different functional groups (two grasses and two non-leguminous forbs) and commonly occurring in the meadow of investigation (e.g. Heinze *et al.* 2016, 2020). The two grasses were *Anthoxanthum odoratum* L. and *Arrhenatherum elatius* (L.) J. Presl & C. Presl. and the forbs were *Achillea millefolium* L. and *Plantago lanceolata* L. Seeds of the four species were collected by hand in the meadow at the field site of the University of Potsdam in summer 2018. For every species, seeds were collected and afterwards pooled from approx. 30 maternal genotypes (spaced at least 2 m apart).

### Plant–soil feedback experiment

To measure PSFs, we used the ‘natural experiment’ approach (Kulmatiski and Kardol 2008).

Following Brandt *et al.* (2014), in early May 2019, 1.5 L species-specific rhizosphere soil was collected from 20 individuals per species, composited into one bulk sample and stored at 4 °C. We used one half of the composite sample as ‘home’ soil (i.e. conspecific soil), and the remaining half to create ‘away’ soils (i.e. soils of the remaining heterospecific species). This mixing procedure is intended to decrease variance in plant responses among individual soil samples and thus increase the likelihood of falsely detecting PSFs (Reinhart and Rinella 2016). However, we were interested in general (rather than within-site variation of) PSF effects and how they are influenced by wind intensity. We therefore consider the mixing approach appropriate (Kulmatiski 2016; Teste *et al.* 2019) especially because soil-handling methods depend on specific research questions and feasibility (Cahill *et al.* 2017; Gundale *et al.* 2019). In total, there were eight soils: four home soils (one for every species) and four away soils that each consisted of equal proportions of soils from the three heterospecific species. To prevent potential differences in soil nutrient availability among the eight soils, an autoclaved soil:sand mixture (5 times within 24 h; 20 min, 121 °C) was inoculated in a ratio of 9:1 with the different home and away soils (i.e. 90 % soil:sand mixture and 10 % pure home or away soil; see also Brinkman *et al.* 2010). The soil:sand mixture consisted of a 1:1 mixture of purchased sand (grain size: 2 mm; Brun & Böhm; Potsdam, Germany) and sieved (mesh size: 5 mm) field soil. The field soil was collected from the same meadow at the field site of the University of Potsdam by removing the aboveground vegetation and collecting the topsoil (25 cm) in various plots.

The inoculated soils were filled into pots (Deepots D25L: volume 0.41 L; height 25 cm; diameter 5 cm; Stuewe & Sons; USA). These pots were individually placed in sterile plastic saucers and received an additional layer (1 cm) of sterilized sand on top to prevent cross-contamination (e.g. Heinze *et al.* 2015; Heinen *et al*. 2018). In April 2019, seeds of the four species were surface-sterilized (3 min in 7 % sodium hypochlorite solution) to reduce microbial contamination. Afterwards, seedlings were germinated on sterilized sand in sterile plastic chambers (32 cm × 50 cm × 14 cm; Meyer; Germany) in a greenhouse (min/max: temperature 15 °C/25 °C; relative humidity 33 %/90 %; additional light: 140 μmol s^−1^ m^−2^; 12/12 h light/dark) at the University of Potsdam. In early May 2019, 2-week-old, similar-sized seedlings within each species were transplanted into the prepared pots, one individual seedling per pot. To ensure the survival of the young seedlings, plants were grown for 1 week in the greenhouse after transplanting. Seedlings that died during this week were replaced. After this establishing phase, pots were moved from the greenhouse to the meadow at the field site and positioned in the prepared wind intensity treatment plots (discussed subsequently).

### Wind treatment

**Figure 1.**
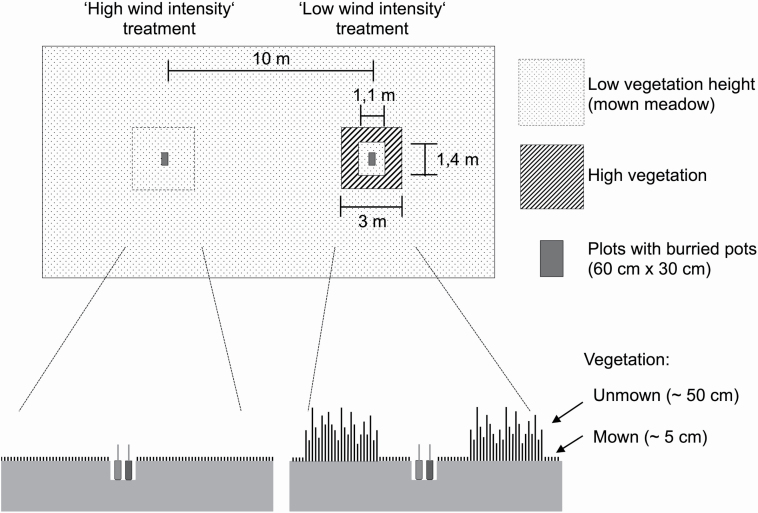
Conceptual figure of the experimental set-up and design. In the meadow, three sites were randomly chosen. These three sites were spaced >50 m apart from each other. Two paired plots (60 cm × 30 cm) 10 m apart were established within each of the three sites. Pots with experimental plants were buried (25 cm deep) in the paired plots to match the soil surface and thus to mimic natural growth (i.e. wind-) conditions in the meadow. For further details see description in the material and methods section.

To investigate the effect of wind intensity on root morphological traits and potentially associated impacts on the outcome of PSFs under natural conditions, we established two wind intensity treatments directly in the meadow. Three sites were chosen in the meadow at the field site of the University of Potsdam. Sites were spaced >50 m apart from each other. Within these three sites two paired plots (60 cm × 30 cm) 10 m apart were established (Fig. 1). In the paired plots, the pots were buried (25 cm depth) to match the level of the soil surface and thus to mimic natural growth conditions and wind exposure in the meadow.

The meadow was mown just before the experiment and afterwards every 2 weeks. In one of the paired plots, neighbouring vegetation was mown (ca. 5 cm) according to the usual mowing regime of the meadow, whereas vegetation around the second paired plot (3 m × 3 m) was left standing (mean vegetation height was ~50 cm; Fig. 1). The mown plots that allowed the wind to fully reach the experimental plants are referred to as ‘high wind intensity’ treatment, whereas the plots with the high vegetation surrounding are referred to as ‘low wind intensity’ treatment throughout the manuscript. To prevent differences in light conditions (i.e. potential shading effects) and microclimate between the high and low wind intensity treatments, the direct neighbouring vegetation (40 cm) of the low wind intensity plots was cut to 5 cm every 2 weeks. This made the vegetation height similar and prevented competition for light between experimental plants and surrounding vegetation.

### Experimental setup

The experiment was performed from early May to mid-June 2019 when wind intensity is usually higher than in mid-summer (average monthly wind intensity based on daily measures from 1998 to 2018: May: 0.719 ± 0.015 m s^−1^; August: 0.523 ± 0.013 m s^−1^ data obtained from the weather station of the University of Potsdam). Furthermore, we chose this early phase of the vegetation period because herbivory intensity by aboveground insects can be expected to be low (see Brown *et al.* 1987) since the cooler temperatures of late spring retard insect development (Bale *et al.* 2002) and feeding (Lemoine *et al.* 2014; Birkemoe *et al.* 2016).

Within the paired wind intensity plots the pots were arranged in a randomized block design and spaced ca 15 cm apart to prevent shading effects. Each plot contained three replicates of each species × soil combination, resulting in a total of 144 pots (3 sites × 2 wind intensities × 4 species × 2 soils × 3 replicates). To reduce potential differences in wind and microclimate between sites within the meadow the pots were shifted between the three sites every second week according to their wind treatment. To prevent differences in wind exposure between plants within the blocks/plots, we re-randomized the pots in the course of the shifting event every second week. During the experiment plants were watered every week with tap water.

### Measurements

To test for differences in wind intensities between the ‘high’ and ‘low’ wind intensity plots, during the experiment, we used anemometer with logger function (Profi-Wind gauge, Bresser GmbH, Germany) in all wind treatment plots at 20 cm height. Furthermore, we used HOBO Pro v2 data loggers (Onset Computer, MA, USA) to measure air temperature and relative air humidity at 20 cm height continuously to test for potential differences in abiotic conditions between the ‘high’ and ‘low’ wind intensity plots. We measured wind speed, air temperature and humidity at 20 cm to obtain data that reflect growth conditions for young plants near the soil surface.

Nutrient differences among soil types were tested using chemical digestion and photometric methods according to Heinze *et al.* (2017), but no differences were found among species or between ‘home’ and ‘away’ soil within species (Table 1).

**Table 1.** Soil characteristics for the four ‘home’ and four ‘away’ soils used in the experiment. Soils contained 90 % sterilized soil:sand mixtures (1:1) inoculated with 10 % ‘home’ or ‘away’ soil of the four plant species. For further details see ‘Material and Method’ section. According to Heinze *et al.* (2016) and Heinze and Joshi (2018)  *P*-values represent results of ANOVAs testing for differences between soils. Data represent mean ± SE, with *n* = 9 for plant-available ammonium (NH_4_^+^) and phosphorus (P); and *n* = 6 for plant-available nitrate (NO_3_^−^), total nitrogen (N) and total P.

	*Anthoxanthum odoratum*		*Arrhetaherum elatius*		*Achillea millefolium*		*Plantago lanceolata*		
Soil characteristics	‘home’	‘away’	‘home’	‘away’	‘home’	‘away’	‘home’	‘away’	*P*
pH	6.99	6.96	7.01	6.99	6.98	7.0	6.96	6.99	
NH_4_^+^ (mg kg^−1^)	12.32 ± 0.54 **a**	12.67 ± 0.43 **a**	12.98 ± 0.40 **a**	12.83 ± 0.48 **a**	12.77 ± 0.44 **a**	12.61 ± 0.39 **a**	12.58 ± 0.51 **a**	12.69 ± 0.49 **a**	n.s.
NO_3_^−^ (mg kg^−1^)	23.18 ± 0.54 **a**	22.76 ± 0.55 **a**	22.17 ± 0.57 **a**	22.31 ± 0.39 **a**	22.18 ± 0.39 **a**	22.91 ± 0.42 **a**	21.95 ± 0.62 **a**	22.59 ± 0.43 **a**	n.s.
Total N (g kg^−1^)	0.44 ± 0.09 **a**	0.46 ± 0.11 **a**	0.46 ± 0.07 **a**	0.47 ± 0.09 **a**	0.46 ± 0.06 **a**	0.46 ± 0.08 **a**	0.42 ± 0.07 **a**	0.45 ± 0.08 **a**	n.s.
P (mg kg^−1^)	1.22 ± 0.03 **a**	1.24 ± 0.05 **a**	1.25 ± 0.04 **a**	1.22 ± 0.04 **a**	1.23 ± 0.04 **a**	1.23 ± 0.03 **a**	1.22 ± 0.05 **a**	1.24 ± 0.03 **a**	n.s.
Total P (g kg^−1^)	0.31 ± 0.04 **a**	0.30 ± 0.03 **a**	0.30 ± 0.03 **a**	0.29 ± 0.04 **a**	0.31 ± 0.04 **a**	0.30 ± 0.05 **a**	0.29 ± 0.03 **a**	0.30 ± 0.05 **a**	n.s.

After 7 weeks of different wind exposure, we checked experimental plants for potential damages by aboveground insect herbivores by visual assessment as described in Heinze *et al.* (2019). Briefly, we visually estimated biomass removal by aboveground insect herbivores (in percent severity; see also, e.g. Johnson *et al.* 2016) at 10 randomly chosen leaves per individual plant. Furthermore, we determined the proportion of damaged leaves by counting the number of damaged as well as total leaves (incidence) on each experimental plant (see Russell *et al.* 2010). Both, severity and incidence were then used to assess the shoot biomass removal by herbivores for whole experimental plants according to Smith *et al.* (2005).

Afterwards, shoots were harvested and roots were washed. To investigate whether wind affects root morphological traits of plants, a subset of individuals of each species (3 replicates—one randomly chosen per site—per soil and wind treatment; i.e. 48 samples in total) was analysed. To determine root diameter and length, a representative subsample (max diameter: 0.56 mm) of the whole root system of each plant was analysed using the WinRhizo scanner-based system (Regents Instruments, Inc., Canada). Afterwards roots were dried (48 h, 80 °C) and weighed to obtain root mass of the subsample. Specific root morphological traits (except average diameter, AD) were calculated according to Ryser and Lambers (1995) and Wright and Westoby (1999): specific root length (SRL; cm mg^−1^), specific root surface area (SRSA; cm^2^ mg^−1^) and root tissue density (RTD; mg cm^−3^). To calculate RTD, we summed the volume of 0.1 mm diameter classes as recommended by Rose (2017). Shoot and root biomass of all experimental plants was dried (48 h, 80 °C) and weighed to assess root:shoot ratio.

### Statistical analysis

All analyses were performed in R version 3.1.2 (R Development Core Team 2014). Prior to analysis, residuals were checked for homogeneity of variance and tested for normality.

PSFs were calculated as log(homeA/awayA), where homeA is the biomass of species A with its own soil biota and awayA is the biomass of species A with soil biota of the three remaining heterospecific species. This allows directly comparing positive and negative feedback effects (see Brinkman *et al.* 2010). Here PSFs were calculated pairwise per block (i.e. replicate; see e.g. Heinze *et al.* 2016) for total biomass separately for the low and high wind intensity plots.

To test whether wind intensity affected biomass production and allocation as well as root morphological traits for the four species on the different soils, we performed ANOVAs. The model included the predictors ‘species’ (*A. odoratum*, *A. elatius*, *A. millefolium*, *P. lanceolata*), ‘soil biota’ (‘home’ vs. ‘away’ soils) and ‘wind treatment’ (‘low’ vs. ‘high’ wind intensity) as well as their interactions and tested their effects on biomass production (shoot, root, total), root:shoot ratio as well as root morphological traits (SRL, SRSA, AD and RTD).

Using the same model, without the factor ‘soil biota’, we furthermore tested whether wind intensity affected the outcome of PSFs. Afterwards, differences in biomass production, PSFs and root morphological traits between ‘low’ and ‘high’ wind intensity and biomass production on the different home and away soils at the different wind intensities were tested using two sample *t*-tests for every species. For each species, we used one-sample *t*-test to assess whether PSFs differed from zero (i.e. were significantly positive or negative).

Additionally, we investigated whether herbivory (i.e. estimated shoot biomass removal) differed between: the two wind levels, species and soil biota (as well as their interactions) using the same linear model as described earlier.

## Results

### Abiotic conditions

Wind intensity significantly differed between both wind treatments (Table 3). In the regularly mown (i.e. ‘high wind intensity’) sites wind intensity was on average 0.698 ± 0.073 m s^−1^, whereas the high vegetation decreased wind intensity on average by 70 % in the ‘low wind intensity’ sites (Table 3). In contrast, the different vegetation surroundings (i.e. wind treatments) did not affect average air temperature and relative air humidity (Table 3).

**Table 2. Summary:** of ANOVA results for herbivory, biomass and root morphological traits. The ANOVAs tested effects for (a) species (*A. odoratum*, *A. elatius*, *A. millefolium*, *P. lanceolata*) and (b) functional group (‘grasses’ vs. ‘forbs’) with soil biota (‘home’ vs. ‘away) and wind intensity (‘low’ vs. ‘high’) and their interactions on damages by herbivores (Herbivory; i.e. estimated shoot biomass removal), biomass production (shoot, root and total biomass as well as root:shoot ratio) and root morphological traits [specific root length (SRL), specific root surface area (SRSA), average diameter (AD) and root tissue density (RTD)]. Significant effects (*P* < 0.05) are reported in bold and marginal significant effects (*P* < 0.1) in italics.

				Biomass									Root traits							
		Herbivory		Shoot		Root		Total		Root:shoot			SRL		SRSA		AD		RTD	
	df	*F*	*P*	*F*	*P*	*F*	*P*	*F*	*P*	*F*	*P*	df	*F*	*P*	*F*	*P*	*F*	*P*	*F*	*P*
(a)																				
Species (S)	3	3.44	**0.020**	8.66	**<0.001**	13.24	**<0.001**	12.46	**<0.001**	8.59	**<0.001**	3	0.89	0.461	1.15	0.349	2.44	*0.089*	2.81	*0.073*
Soil biota (SB)	1	0.04	0.843	0.01	0.983	0.45	0.505	0.14	0.711	1.97	0.163	1	0.02	0.892	0.03	0.862	0.38	0.546	0.31	0.584
Wind (W)	1	2.55	0.113	2.67	0.112	3.33	*0.071*	3.71	*0.058*	2.64	0.139	1	7.65	**0.011**	6.19	**0.020**	13.63	**0.001**	29.42	**<0.001**
S × SB	3	0.01	0.989	1.35	0.263	0.66	0.577	1.08	0.362	0.94	0.424	3	0.11	0.955	0.13	0.944	0.30	0.823	0.35	0.792
S × W	3	1.57	0.157	0.12	0.946	0.27	0.850	0.16	0.920	1.11	0.348	3	0.32	0.811	0.57	0.642	2.29	0.104	2.38	0.108
SB × W	1	0.01	0.915	0.13	0.721	0.96	0.331	0.57	0.454	0.16	0.700	1	0.18	0.677	0.17	0.686	0.12	0.728	0.14	0.709
S × SB × W	3	0.14	0.931	2.23	*0.089*	1.59	0.196	2.30	*0.082*	0.31	0.815	3	0.19	0.899	0.13	0.944	0.76	0.526	0.29	0.832
Residuals	124											32								
(b)																				
Functional group (F)	1	3.53	**0.063**	2.96	*0.088*	23.60	**<0.001**	13.09	**<0.001**	22.78	**<0.001**	1	1.74	0.197	0.87	0.357	2.75	0.107	2.14	0.156
Soil biota (SB)	1	0.01	0.975	0.08	0.773	0.16	0.693	0.01	0.951	1.53	0.218	1	0.06	0.804	0.11	0.739	0.31	0.583	0.16	0.691
Wind (W)	1	2.54	0.114	1.48	0.225	2.29	0.133	2.24	0.137	2.37	0.167	1	9.91	**0.004**	7.85	**0.009**	12.97	**0.001**	31.35	**<0.001**
F × SB	1	0.02	0.900	2.21	0.140	1.27	0.262	2.03	0.158	0.42	0.520	1	0.02	0.903	0.22	0.642	0.58	0.453	0.07	0.786
F × W	1	1.91	0.192	0.01	0.925	0.09	0.768	0.01	0.912	2.45	0.166	1	0.90	0.351	0.91	0.346	3.27	*0.080*	3.35	*0.092*
SB × W	1	0.11	0.741	0.23	0.632	1.06	0.306	0.69	0.409	0.17	0.682	1	0.35	0.560	0.46	0.503	0.00	0.958	0.00	0.995
F × SB × W	1	0.04	0.839	5.89	**0.017**	4.65	**0.034**	6.28	**0.015**	0.44	0.506	1	0.05	0.817	0.04	0.834	0.23	0.636	0.04	0.836
Residuals	140											40								

**Table 3.** Abiotic conditions in the ‘low wind intensity’ (i.e. high vegetation surrounding) and ‘high wind intensity’ (i.e. mown vegetation) treatment. Data represent average daily means ± SE (49 days). *P* values represent results from t-test analyses that tested for differences between the two wind intensity treatments.

	Low wind intensity	High wind intensity	
	(high vegetation height)	(low vegetation height)	*P*
Wind intensity (m s^−1^)	0.208 ± 0.032	0.698 ± 0.073	<0.001
Air temperature (°C)	22.94 ± 0.595	22.88 ± 0.639	n.s.
Rel. air humidity (%)	68.82 ± 1.459	66.28 ± 1.683	n.s.

### Impact of wind intensity on biomass allocation and biomass production

**Figure 2.**
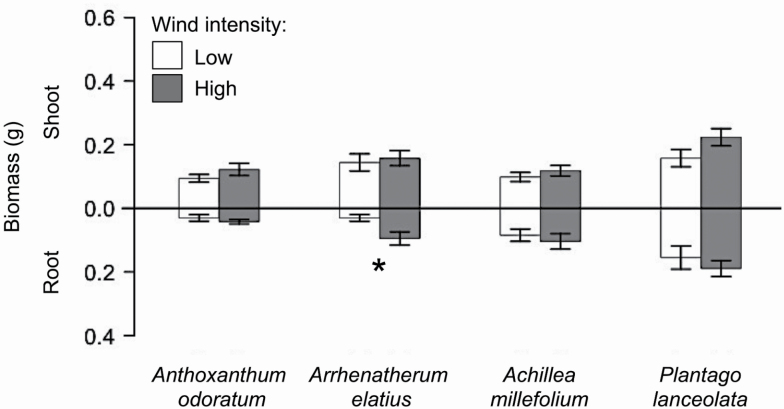
Shoot (top) and root (bottom) biomass of *Anthoxanthum odoratum*, *Arrhenatherum elatius*, *Achillea millefolium* and *Plantago lanceolata* when grown under low (white) and high (grey) wind intensity. Data represent mean ± SE (*n* = 18). Significant differences in biomass production between the two wind treatments are indicated by asterisks: *P < 0.05.

Wind intensity neither affected overall biomass allocation (i.e. root:shoot ratio; *F*_1,124_ = 2.64; *P* = 0.139; Table 2a), nor the root:shoot ratio between the four species (species × wind: *F*_3,124_ = 1.11; *P* = 0.348; Table 2a; see Fig. 2). However, high wind intensity slightly increased root biomass production (*F*_1,124_ = 3.33; *P* = 0.071; Table 2a) and thus total biomass (*F*_1,124_ = 3.71; *P* = 0.058; Table 2a), but only because of effects on one grass species (*A. elatius*; see Fig. 2). In contrast, shoot biomass production was not affected by wind intensity (*F*_1,124_ = 2.67; *P* = 0.112; Table 2a).

### Effects of wind intensity on herbivory

Overall, there was only weak damage by insect herbivores (average shoot biomass removal by insect herbivores across species: 0.44 ± 0.06 %) with no differences found between the two wind levels and among ‘home’ and ‘away’ soil within species at the two wind levels (Table 2).

### Effects on root morphological traits

**Figure 3.**
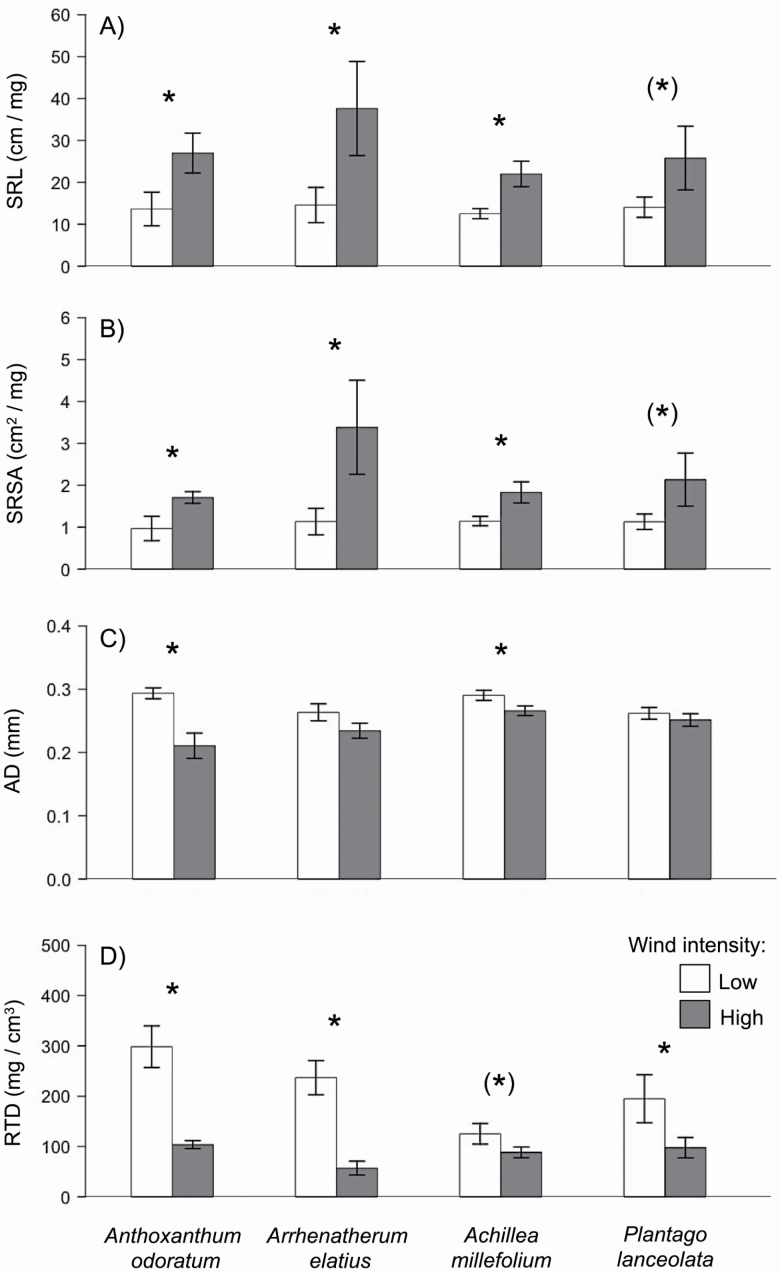
Specific root length (SRL; A), specific root surface area (SRSA; B), average root diameter (AD) and root tissue density (RTD) of *Anthoxanthum odoratum*, *Arrhenatherum elatius*, *Achillea millefolium* and *Plantago lanceolata* when grown under low (white) and high (grey) wind intensity. Because soils (i.e. home and away) had no different effect on root morphological traits (Table 2a) data of the two soils were combined. Data represent mean ± SE (*n* = 6). Significant differences in biomass production between the two wind treatments are indicated by asterisks: ^(^* ^)^*P* < 0.1; *P < 0.05; ***P < 0.001.

Wind intensity influenced fine-root morphology of the four species (SRL: *F*_1,32_ = 7.65, *P* = 0.011; SRSA: *F*_1,32_ = 6.19, *P* = 0.020; AD: *F*_1,32_ = 13.63, *P* = 0.001; RTD: *F*_1,32_ = 29.42, *P* < 0.001; Table 2a). Across species, SRL and SRSA were higher in the high wind intensity plots compared with low wind intensity plots, whereas AD and RTD showed reverse pattern (Table 2; Fig. 3)—i.e. high wind intensity resulted in thinner roots with increased length and surface area per unit biomass investment. These wind effects were in general more pronounced for grasses than for forbs (SRL: *F*_1,40_ = 9.91, *P* = 0.004; SRSA: *F*_1,40_ = 7.85, *P* = 0.009; AD: *F*_1,40_ = 12.97. *P* = 0.001; RTD: *F*_1,40_ = 31.35. *P* < 0.001; Table 2b). Grasses showed a stronger increase in SRL and SRSA (SRL: +136 %; SRSA: + 158 %) and a stronger decrease in AD and RTD (AD: −22 %; RTD: −71 %) under high wind intensities when compared with forbs (SRL: +80 %; SRSA: +74 %; AD: −8 %, RTD: −42 %; see Fig. 3). Soils (i.e. home and away) had no effect on root morphological traits (Table 2).

### PSF effects under different wind intensities

**Figure 4.**
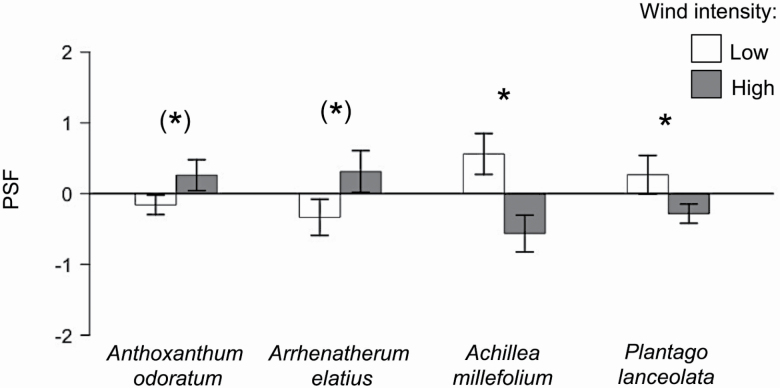
Plant–soil feedback (PSF; log total dry home vs. away biomass ratio) of *Anthoxanthum odoratum*, *Arrhenatherum elatius*, *Achillea millefolium* and *Plantago lanceolata* when grown under low (white) and high (grey) wind intensity. Data represent mean ± SE (*n* = 9). Differences in biomass between the wind treatments are indicated by asterisks: ^(^* ^)^*P* < 0.1; *P < 0.05.

**Fig. 5.**
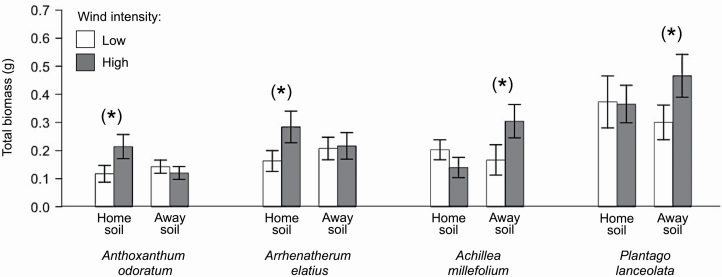
Total biomass of *Anthoxanthum odoratum*, *Arrhenatherum elatius*, *Achillea millefolium* and *Plantago lanceolata* when grown on ‘home’ (left bars) and ‘away’ (right bars) soils under low (white) and high (grey) wind intensity. Data represent mean ± SE (*n* = 9). Differences in biomass between the wind treatments are indicated by asterisks: ^(^* ^)^*P* < 0.1.

Wind intensity influenced the outcome of PSFs for total plants (shoot and root), but these effects differed among the four plant species (species × wind: *F*_3,64_ = 4.61, *P* = 0.004; Table 4; Fig. 4) by slightly affecting total biomass production differently for the four species on home vs. away soils (species × soil × wind: *F*_3,124_ = 2.30, *P* = 0.082; Table 2a; Fig. 5). These wind effects on home and away soils strongly differed between functional groups (PSFs: *F*_1,68_ = 12.74, *P* < 0.001; Table 4; Fig. 4; total biomass: *F*_1,140_ = 6.28, *P* = 0.015; Table 2b; Fig. 5). Albeit the strength of PSFs was weak in general (i.e. PSFs were not significantly positive or negative), increasing wind intensity changed the direction of PSFs in all four species as well as for grasses vs. forbs (Fig. 4). High wind intensity slightly increased PSFs for the two grass species, from weak negative at low wind intensity to weak positive PSFs at high wind intensity (Fig. 4). This was because in the two grass species high wind intensity increased biomass production on home soils, whereas total biomass remained similar in away soils (Fig. 5). In contrast, PSFs for the two forbs significantly decreased from positive PSFs to negative PSFs (Fig. 4), because high wind intensity increased biomass production only on away soils for these two forbs (Fig. 5).

**Table 4.** Summary of ANOVA results for plant-soil feedback effects (PSF; log total dry home vs. away biomass ratio). The ANOVAs tested effects for (a) species (*A. odoratum*, *A. elatius*, *A. millefolium*, *P. lanceolata*) and (b) functional group (‘grasses’ vs. ‘forbs’) with wind intensity (‘low’ vs. ‘high’) and their interactions on PSFs for shoot, root and total plants. Significant effects (*P* < 0.05) are reported in bold.

		PSF					
		Shoot		Root		Total	
	df	F	*P*	F	*P*	F	*P*
(a)							
Species (S)	3	2.02	0.114	0.35	0.789	0.63	0.599
Wind (W)	1	1.84	0.177	1.40	0.131	1.89	0.171
S × W	3	5.29	**0.002**	3.98	**0.009**	4.61	**0.004**
Residuals	64						
(b)							
Functional group (F)	1	2.36	0.118	0.65	0.420	1.63	0.204
Wind (W)	1	1.85	0.175	1.44	0.133	1.95	0.164
F × W	1	14.06	**< 0.001**	7.69	**0.006**	12.74	**< 0.001**
Residuals	68						

## Discussion

The overarching goal of this study was to investigate the impact of wind intensity on biomass allocation and fine root morphological traits of grassland plants as well as potential changes in the outcome of plant–soil feedbacks under natural field conditions. Though we could not detect an effect of wind intensity on root:shoot ratio, we found uniform effects on root morphological traits irrespective of plant species. When grown under high wind intensity plant roots—especially of the two grasses—showed lower AD and RTD and higher SRL and SRSA compared with low wind intensity. Plant biomass production on home vs. away soils—i.e. PSF effects—was also influenced by wind intensity with stronger effects for the two forbs when compared with the grass species. As home and away soils did not differ in plant-available nor total nutrients these results suggest that wind-induced changes in root morphological traits, that shape interactions with soil biota (Friesen *et al.* 2011; Bergmann *et al.* 2016; Rutten and Gòmez-Aparicio 2018), have the potential to alter the outcome of PSFs.

### Wind effects on biomass allocation

It is widely acknowledged that wind, as a mechanical aboveground stimulus, influences the allocation of assimilates from shoots to roots (Cleugh *et al.* 1998), leading to increased root growth (Nicoll and Ray 1996) and root:shoot ratio in many plants (Poorter *et al.* 2012; Gardiner *et al.* 2016; Feng *et al.* 2019). Most studies on wind effects on biomass allocation focus on woody plant species (see Gardiner *et al.* 2016), while few studies found similar pattern in herbaceous plants (Whitehead 1962; Retuerto and Woodward 1992; Henry and Thomas 2002). In contrast to these previous findings, root:shoot ratio was not affected by wind intensity in any of our grassland species. This might because most of the previous studies used electric fans and exposed plants to abnormally high wind speed, with average wind speed ranging from 3 to 10 m s^−1^ (see e.g. Whitehead 1962; Henry and Thomas 2002; Feng *et al.* 2019), whereas plants in our experiment grew at soil level and were exposed to natural wind speed, that generally decreases with decreasing height (Goudriaan 1977; Speck 2003). Furthermore, we included a realistic control (i.e. we did not fully exclude wind in our experiment) in contrast to most other studies (see Poorter *et al.* 2012; Gardiner *et al.* 2016). Average wind speed in the experiment was 0.698 m s^−1^ at the regularly mown plots and 0.208 m s^−1^ in plots with high surrounding vegetation. Hence, differences in wind speed in our experiment might have been too small to induce changes in biomass allocation reported for less realistic comparisons. Although our study reveals that, under natural conditions, wind effects might not be crucial for biomass allocation of young plants near ground level it is possible and likely that such effects will become more important with increasing plant height and wind speed (Goudriaan 1977; Speck 2003).

### Root morphological traits at the two wind intensities

Past studies on the effects of wind on roots mostly focussed on root system architecture in the context of plant anchorage and stability (Stokes *et al.* 1995; Danjon *et al.* 2005; Tamasi *et al.* 2005; Stofko and Kodrik 2008) particularly in woody species. Beside root system architecture, characteristics of individual roots (i.e. morphology) are also important for anchorage (Ennos and Fitter 1992; Goodman *et al.* 2001; Burylo *et al.* 2009) but these have been rarely linked to wind effects (Goodman and Ennos 1997) especially in herbaceous plants. In our study, even a small increase in wind speed (at generally low levels of wind) resulted in a significant increase in SRL and SRSA as well as a decrease in AD and RTD in the young plants of all four grassland species. Thinner roots with increased SRL and SRSA increase plant–soil contact. Hence, our results indicate that young plants when exposed to high wind intensity changed morphology of their fine roots in ways expected to strengthen anchorage, as also found for young trees by Tamasi *et al.* (2005). A lower RTD under high wind intensity implies that these plants invest less root biomass to achieve given levels of surface area and length and are hence greater root–soil contact at less cost. This could not only lead to a better anchorage but increase relative growth rate and enhance resource acquisition (see e.g. Waddell *et al.* 2017). Our results might also indicate that in addition to effects on anchorage, increasing wind intensity induces plastic changes in root morphology linked to the resource-use strategy of young plants.

In our experiment, changes in fine root morphological traits were more pronounced for grasses when compared with forbs. The stronger increase in SRL and SRSA and the stronger decrease in AD and RTD under high wind intensities compared with forbs might be due to the fact that grasses taller in this experiment (J. Heinze, pers. comm.) and hence might have been exposed to higher wind speeds (Goudriaan 1977; Speck 2003).

Furthermore, wind causes various kinds of damage in leaves, e.g. by the rubbing of adjacent leaves (Grace 1988). Studies in grasses [*Festuca arundinacea* Schreb. and *Molinia cearula* (L.) Moench] revealed that wind ruptures epidermal cells and cracks the cuticle (Thompson 1974, Pitcairn and Grace 1984). Such mechano-stimulated damage in plants induce changes in concentrations of phytohormones like auxin, ethylene, cytokinins and abscisic acid that regulate plant growth and development (‘thigmomorphogenesis’, e.g. Jaffe 1973; Chehab *et al.* 2009) including the elongation and branching of roots (Lee *et al.* 2018; Lymperopoulos *et al.* 2018) and thus root morphology. Therefore, although generally low, it is possible that higher wind speeds could have changed fine-root morphology due to more leaf damage.

Beside the involvement in anchorage, root morphological traits are also important for the uptake of limiting resources such as nitrogen, phosphorus and water (Bardgett *et al.* 2014; Reich 2014). High wind intensity was found to increase the uptake of carbon dioxide (CO_2_) and assimilation rate in plants (Wadsworth 1959; Retuerto and Woodward 1992). Therefore, a change towards thinner roots with increased specific surface area at high wind intensity might be an adaption to increased nutrient requirements for assimilation. In accordance with this, the decrease in RTD might also indicate that plants become more nutrient acquisitive. Though this may be a possible further explanation for the observed pattern, the impact of wind on CO_2_ uptake should not be overestimated (Grace 1988) since our results show that wind only slightly increased total biomass production.

However, as our study was not designed to explore particular mechanisms behind wind-induced changes in root morphological traits we did not measure, e.g. concentrations of phytohormones or CO_2_, rates of CO_2_ uptake or photosynthesis, nutrient contents of roots and shoots or other abiotic factors. Hence, suggestions about specific mechanisms involved are merely speculative. Nevertheless, our study reveals that wind intensity does influence root morphological traits of grasslands plants, with high wind intensity increasing SRL and SRSA and decreasing AD and RTD.

### Wind and PSF effects

In addition to their importance for anchorage and nutrient uptake, fine root morphological traits are relevant for soil processes such as decomposition and interactions with soil biota (Friesen *et al.* 2011; Bardgett *et al.* 2014). Therefore, wind-induced changes in root morphology may influence plant–soil interactions and thus the outcome of PSFs, as found in our experiment. In this study, PSFs effects were generally weak but differed between low and high wind intensity for all species. However, changes in PSFs in response to wind were particularly pronounced between functional groups, because PSFs for grasses increased under high wind intensity, whereas PSFs of forbs decreased. Although these PSF effects partly suggest strong effects of wind, especially for forbs, a closer view on biomass production, however, revealed only marginal differences between the two wind treatments.

At low wind intensity total biomass production on home and away soils was similar for all four species indicating that PSF effects for the young plants were neutral in this wind treatment. This was surprising because two of these species (*A. odoratum* and *P. lanceolata*) showed remarkably negative and positive PSFs in an earlier experiment in the same meadow (Heinze *et al.* 2020). An advantage of the inoculation method (Brinkman *et al.* 2010) is that home and away soils neither differed in plant-available nor total nutrients in the beginning of the experiment. Hence, potential differences in biomass production on the different soils would most probably have been caused by altered soil biota. However, the neutral PSF effects (i.e. no significant positive or negative PSF) in our experiment suggest that effects of soil biota on the young plants (i.e. their interaction) were not distinct enough to cause effects on plant growth. Because the strength of PSFs was found to increase over time (Hawkes *et al.* 2013) and to be a function of length of the test phase (Kardol *et al.* 2013), the neutral PSFs in this study (as opposed to our earlier study) might originate from the shorter growth period.

High wind intensity resulted in increased biomass production on home soils for the two grasses and on away soils for the two forbs. In contrast, total biomass remained similar between low and high wind intensity on away soils for grasses and home soils for forbs, thus influencing the outcome of PSF (i.e. biomass production on home relative to away soils), especially between functional groups. Home and away soils showed similar nutrient content and did not affect root morphological traits, but impacted biomass production differently according to wind level. This indicates that wind-induced changes in fine root morphological traits have the potential to affect biomass production. Such changes in fine root morphological traits could have influenced both nutrient uptake and interactions with soil biota (Friesen *et al.* 2011; Bardgett *et al.* 2014). However, roots were thinner and showed an increased specific root surface area in both, home and away, soils at high wind intensity. Therefore, it is unlikely that the difference in biomass production was caused by the mere abiotic effect of increased resource uptake efficiency, potentially mediated by, e.g. increased SRL (Thorne and Frank 2009), because both soils were similar in nutrient content. It seems more likely that intensified root-surface interactions with soil biota are involved (Bardgett *et al.* 2014). For instance, *A. odoratum* exudates more coumarin compared with other species (Tava *et al.* 2001) suggesting that home soil of *A. odoratum* and away soils of the other species (including one-third home soil of *A. odoratum*) contained higher amounts of coumarin. There is evidence that coumarin suppresses soil pathogens and increases beneficial rhizobacteria (Stringlis *et al.* 2018). Therefore, higher biomass production of *A. odoratum*, *A. millefolium* and *P. lanceolata* in home or away soils at high wind intensity might have been caused by more interactions with beneficial soil biota due to higher specific root surface area. The most dominant species in this meadow, *A. elatius*, typically shows neutral PSFs and no benefit from *A. odoratum* soil (see Heinze *et al.* 2016, 2020; Heinze and Joshi 2018). In our experiment, however, *A. elatius* showed the highest biomass on home soil at high wind intensity. We can only speculate whether the increased SRL and SRSA at high wind intensity caused more interactions with species-specific mutualists and thus increased growth on home soil.

We were not able to differentiate mechanistically between beneficial and harmful soil biota in our experiment. However, we show that changes in root morphological traits in general have the potential to influence interactions between plants and soil biota. Furthermore, we suggest that DNA-sequencing techniques of soil micro-organisms will be needed to advance our understanding of the role of soil biota in wind mediated PSF–root-morphology interactions. As wind is an environmental factor typically excluded under greenhouse conditions (Heinze *et al.* 2016; Forero *et al.* 2019) our results provide a potential explanation for reported differences in PSFs between greenhouse and field. Overall, wind-induced changes in PSFs were not very strong in the current short-term study. This is indicated by only marginal trends in biomass production on the different soils. Hence, as wind effects on plants increase with increasing plant height (Goudriaan 1977; Speck 2003), we suggest that the effect of wind-induced changes in root morphological traits on PSFs will differ between shorter and taller plants and generally strengthen with plant height.

## Conclusions

This study appears to be the first to examine effects of wind-induced changes in fine root morphological traits on the outcome of PSFs (i.e. plant–soil interactions). We found wind intensity to influence fine root morphological traits and that a discernible if weak PSF effect changed direction depending on wind intensity. As home and away soils did not differ in nutrient content, our results suggest that wind-induced changes in root morphological traits have the potential to influence the outcome of PSFs (see also Heinze 2020). Our findings are based on one single short-term experiment with four grassland species grown under natural conditions. More work is, therefore, needed to elucidate further the ability of wind-induced changes in root morphology to affect plant–soil interactions. Such experiments could usefully incorporate a wider range of conditions and species (e.g. of different growth forms) and be longer term. Additional measurements on physiological parameters, as suggested earlier, and measurements of the whole root system are also desirable. Furthermore, to validate our finding and to extend them, we suggest future studies test wind effects explicitly on PSFs by manipulating wind intensity in a conditioning phase and testing responses (without wind) in a feedback phase.

Taken together, linking wind-induced changes in fine root morphology to PSF effects represents one step towards a closer understanding of plant–soil interactions under changing environmental conditions.

## Data Availability

Data are available and can be accessed at https://doi.org/10.6084/m9.figshare.12905852.v1.

## Notes

Plants, Ecosystems & Climate. Chief Editor: Mary Heskel
